# Match Rates Between Home Health Assessment and Medicare Claims Data

**DOI:** 10.1001/jamanetworkopen.2026.4788

**Published:** 2026-04-02

**Authors:** Momotazur Rahman, Xiao (Joyce) Wang, Jamie M. Smith, Christopher M. Santostefano, Jeffrey Hiris, Jianhui Xu, Elizabeth M. White, Katherine A. Ornstein

**Affiliations:** 1 Department of Health Services, Policy and Practice, Brown University School of Public Health, Providence, Rhode Island; 2 Dwyer School of Nursing, Widener University, Chester, Pennsylvania; 3 Department of Health Policy and Management, Bloomberg School of Public Health, Johns Hopkins University, Baltimore, Maryland; 4 Johns Hopkins University School of Nursing, Division of Geriatric Medicine and Gerontology, School of Medicine, Department of Health Policy and Management, Bloomberg School of Public Health, Baltimore, Maryland

## Abstract

**Importance:**

The Outcome and Assessment Information Set (OASIS) is essential for measuring home health quality and outcomes, and accurate linkage between OASIS, Medicare enrollment, and claims is crucial for monitoring utilization and informing payment policy. Preliminary documentation suggested a decrease in OASIS–beneficiary match rates beginning in 2019, but the extent and implications of this decrease have not been quantified.

**Objective:**

To quantify annual match rates between OASIS assessments and Medicare enrollment and fee-for-service (FFS) home health claims from 2017 through 2023.

**Design, Setting, and Participants:**

This cohort study used 100% OASIS assessments from 2017-2023 linked to the Master Beneficiary Summary File (MBSF) and FFS home health claims. OASIS assessments included patients receiving home health care in any payer category, while claims analyses were restricted to FFS beneficiaries. Data were analyzed from June 2025 to January 2026.

**Main Outcomes and Measures:**

The yearly number and proportion of OASIS assessments that can be matched to a person in the MBSF and the yearly number and proportion of FFS home health claims that can be matched to an OASIS assessment.

**Results:**

Across 2017-2023, approximately 18 million OASIS assessments and 6 million unique assessed beneficiaries were recorded annually. The proportion of assessments linked to a Medicare beneficiary decreased from 89.8% in 2017 to 76.4% in 2023. Among unmatched assessments, the share with Medicare as the recorded payer increased, while the share with Medicaid decreased. For FFS beneficiaries, the number of claims increased due to payment cycle changes, yet the number of unique FFS claimants decreased from 3 424 394 in 2017 to 2 636 931 in 2023. The match rate of FFS claims to an OASIS assessment decreased from 96.8% to 73.9%, with variation across states; by 2023, all state match rates were below 90%.

**Conclusions and Relevance:**

The findings of this study suggest a reduced ability to accurately attribute OASIS assessments to individual Medicare beneficiaries or matching claims to corresponding OASIS assessments using Centers for Medicare & Medicaid Services (CMS) Research Identifiable Files. Until these issues are addressed by CMS, researchers should cautiously interpret findings utilizing individually linked Medicare claims and OASIS assessment data.

## Key Points

**Question:**

Has the linkage between Outcome and Assessment Information Set (OASIS) assessments and Medicare enrollment and claims remained stable from 2017 to 2023, and what does this suggest for measuring home health use and outcomes?

**Findings:**

In this cohort study of a US national analysis of 18 million OASIS assessments per year, the share linked to a Medicare beneficiary decreased from approximately 90% in 2017 to 76% in 2023, and the proportion of fee-for-service home health claims with a corresponding OASIS assessment decreased from approximately 97% to 74% over the same period.

**Meaning:**

Results of this study suggest that researchers should interpret with caution any analyses linking OASIS with Medicare data unless a corrected beneficiary identifier is made available by the Centers for Medicare & Medicaid Services.

## Teaser

This cohort study quantifies annual match rates between home health assessment and Medicare enrollment available from research files from 2017 through 2023.

## Introduction

Over the past decade, Medicare’s home health benefit has remained a large, stable component of care delivered in the community: more than 12 000 Medicare-certified home health agencies (HHAs) operate nationally and serve millions of beneficiaries each year.^1,2^ These agencies deliver skilled nursing, therapy (physical therapy/occupational therapy/speech), and medical social services in beneficiaries’ homes, supporting both postacute episodes after hospitalization^3,4^ and community-initiated skilled care that can substitute for more intensive institutional care,^5,6^ and better align with patient preferences. In spending terms, home health totals approximately $460 per traditional fee-for-service (FFS) Medicare enrollee per year, approximately 4% to 5% of total FFS program spending per enrollee, and both levels have been fairly stable over the past decade.^7^ Another well-known feature of home health care is the substantial geographic variation^5,8^ that accounted for a large portion of the geographic variation in traditional Medicare spending.^9^ Taken together, these facts make it essential to accurately measure who receives home health care, how it is delivered, and what outcomes and quality patients experience, especially when evaluating policy reforms and system shocks that can alter care patterns.

The Outcome and Assessment Information Set (OASIS) is a standardized clinical assessment that all Medicare-certified home health agencies are required to complete, making it the primary data source for assessing home health care quality. Unlike claims, OASIS includes detailed measures of function, cognition, and social supports that are important for risk adjustment and evaluating outcomes and care transitions.^10,11^ Additionally, due to incomplete Medicare Advantage (MA) home health encounter data,^12^ OASIS may be the only reliable source of home health data for MA enrollees.^13^

To support research and program monitoring, OASIS records are processed within Centers for Medicare & Medicaid (CMS) systems and released as Research Identifiable Files through Chronic Condition Warehouse (CCW)/Research Data Assistance Center (ResDAC), where analyses typically require linkage to Medicare enrollment and claims using encrypted beneficiary identifiers. In principle, linkage should be highly complete because OASIS assessments are submitted for each home health episode^14^ and are intended to correspond to services ultimately billed to Medicare.^15^ Although OASIS provides clinical information that is not reported on claims, detailed documentation related to services billed to Medicare, such as clinician visit timing and minutes, can only be found on claims. More accurate care utilization or patient outcomes for FFS beneficiaries also require linkage to other claims sources, such as inpatient and outpatient claims. As a result, researchers commonly use the beneficiary identifier crosswalk to connect OASIS assessments to the Master Beneficiary Summary File (MBSF) and FFS home health claims, enabling measurement of home health use, timing, and outcomes. However, linkage performance depends on the integrity of the identifier mapping between OASIS person identifiers and Medicare beneficiary identifiers maintained in the research files.

In 2019, the CCW Data Dictionary for OASIS noted a source system change followed by an 8% to 12% decrease in match rates between OASIS resident identifiers and Medicare beneficiary identifiers.^16^ Although this change and resulting mismatch rates were noted in technical materials, this degradation had not been widely disseminated to end users of OASIS-linked research files or to the health services research community at large. Additionally, it is unclear whether the mismatch rates changed over time. CMS periodically transitions between Medicare administrative contractors responsible for processing FFS claims and the encoding process intended to deidentify individual data before making files available to researchers.^17^ Recent linkage performance is critical to clarify given essential changes that occurred in home health care delivery during COVID-19 and the introduction of a major reform in home health care payment, the Patient-Driven Groupings Model (PDGM), in January 2020.^18,19^ Deteriorating linkage could lead to underascertainment of home health use and biased estimates of trends and outcomes in studies relying on OASIS-claims linkages.

The objective of this study was to quantify annual match rates between OASIS assessments and Medicare enrollment as well as between FFS home health claims and OASIS assessments using the beneficiary identifiers currently available in OASIS research files from 2017 through 2023. We examined changes over time and variation by state and OASIS-reported payer source to inform interpretation of research on home health use, outcomes, and trends that rely on OASIS-linked data.

## Methods

### Study Oversight and Reporting Guideline

This cohort study was approved by the Brown University Institutional Review Board, which waived the requirement for participant informed consent because it used deidentified secondary data. We report this study in accordance with the Strengthening the Reporting of Observational Studies in Epidemiology (STROBE) reporting guideline.

### Data Sources

We used national Medicare administrative and assessment data from January 1, 2017, through December 31, 2023. Data were analyzed from June 2025 to January 2026.

#### OASIS

OASIS is the primary national assessment system for Medicare-certified HHAs. As a condition of participation, CMS requires agencies to submit OASIS assessments for patients receiving home health care (including MA beneficiaries) at key time points, including start of care, follow-up intervals during an episode, and discharge. OASIS includes information on sociodemographic characteristics, living environment, support systems, clinical status, cognition, functional status, and items related to home health care delivery.

#### Master Beneficiary Summary File

The MBSF is the base file that documents the entire US population enrolled in or entitled to Medicare benefits. The MBSF includes Medicare beneficiary enrollment and demographic information, including an encrypted beneficiary identifier, ZIP code of residence, demographic characteristics, monthly dual-eligibility indicators, and monthly MA enrollment indicators.

#### Medicare FFS Home Health Claims

We used Medicare Standard Analytic Files for HHA claims to measure FFS home health utilization.

### Study Populations

We conducted analyses with 2 complementary populations. First, we used all OASIS assessments to quantify the proportion that could be linked to a Medicare beneficiary in the MBSF. Second, we used the population of beneficiaries with at least 1 FFS HHA claim to quantify the proportion of claims that could be traced to a corresponding OASIS assessment.

### Study Variables

The primary variables were dates that define encounter timing in OASIS or HHA claims. For OASIS, we used the effective date, as well as the start and end date of assessment recertification windows. For HHA, we used the start and end date of HHA claim episodes. The HHA claim episode was defined as a single payment period (ie, 60 days prior to 2020, 30 days starting in 2020 due to the PDGM). To assess the potential payer-related patterns among OASIS assessments unmatched to the MBSF, we used the payer source fields recorded in OASIS (Medicare FFS, MA, and Medicaid). In rare cases where multiple payer sources were recorded, we prioritized categories in the following order: Medicare FFS, MA, and then Medicaid. Because prior validation studies are lacking, we assessed the internal validity of these fields by comparing OASIS-reported payer source with enrollment status in MBSF in the month of the assessment effective date.

### Statistical Analysis

We linked 100% of OASIS assessments from 2017 to 2023 to the MBSF using the encrypted beneficiary identifier available in the research files and calculated, by year, the number of OASIS assessments and the percentage matched to a Medicare beneficiary. These rates were computed regardless of whether the beneficiary was enrolled in FFS Medicare or MA.

Among OASIS assessments that did not link to the MBSF, we examined the payer source recorded in OASIS. Specifically, we calculated and plotted, by year, the distribution of payer source among unmatched assessments (Medicare FFS, MA, and Medicaid) to assess whether the share of unmatched assessments with Medicare as payer source has changed over time.

Among OASIS assessments that could be matched to MBSF, we used MBSF monthly MA indicators to identify OASIS assessments belonging to beneficiaries enrolled in FFS Medicare (ie, not enrolled in MA in the relevant month) and tabulated, by year, the number of OASIS assessments among FFS beneficiaries, and the number of unique FFS beneficiaries with any OASIS assessment.

Separately, we used FFS HHA claims to tabulate, by year, the total number of home health claim records and the unique number of FFS beneficiaries with any home health claims. We assessed whether the decrease in the number of OASIS assessments from FFS beneficiaries as identified by merging to the MBSF was steeper than the decrease in the number of FFS beneficiaries with claims.

We calculated the share of HHA claims that could be matched to an OASIS assessment using 2 approaches. Approach 1 is concordance at the beneficiary-year level. Among FFS beneficiaries with at least 1 HHA claim in a year, we defined concordance as the beneficiary having at least 1 OASIS assessment with an effective date in the same calendar year. Approach 2 is overlap between claims and OASIS episode windows. We defined a claim as matched if there was a 1 or more day overlap between the claim episode window (claim start and end dates) and the OASIS episode window (OASIS start and end dates). Because both windows may span calendar years, overlaps were assessed across the full episode windows rather than restricting to within-year dates.

To assess geographic variation in claims-to-OASIS matching, we calculated state-level match rates using the episode-window overlap definition (approach 2) for 2017 and 2023, assigned by beneficiary state of residence. Analyses were implemented using SAS version 9.4 (SAS Institute).

## Results

From 2017 to 2023, the number of OASIS assessments and the unique beneficiaries in OASIS remained stable (approximately 18 million and 6 million, respectively), but the share of assessments matched to a Medicare beneficiary decreased from 89.8% to 76.4% (Table 1). The stability in OASIS volume alongside decreasing match rates suggests a growing share of assessments that cannot be linked to a Medicare beneficiary using current identifiers.

**Table 1.** Trends in the Number of OASIS Assessments and Proportion That Can Be Matched to Medicare Beneficiaries, 2017-2023^a^

Year	Total count of OASIS assessments	Count of unique individuals with OASIS assessments	OASIS assessments that match to a Medicare beneficiary using the MBSF, %
2017	18 540 333	5 850 134	89.8
2018	18 854 664	5 967 062	88.9
2019	18 848 705	6 055 092	86.6
2020	17 990 191	5 895 600	82.6
2021	18 683 535	6 135 169	80.3
2022	18 211 410	6 045 602	78.9
2023	17 637 253	5 911 369	76.4

Abbreviations: MBSF, Medicare Beneficiary Summary File; OASIS, Outcome and Assessment Information Set.

^a^ OASIS assessments were attributed to a year based on the assessment effective date. The numbers include all individuals included in OASIS, including both Medicare Advantage enrollees and fee-for-service beneficiaries, as well as non-Medicare beneficiaries.

**Figure 1.**
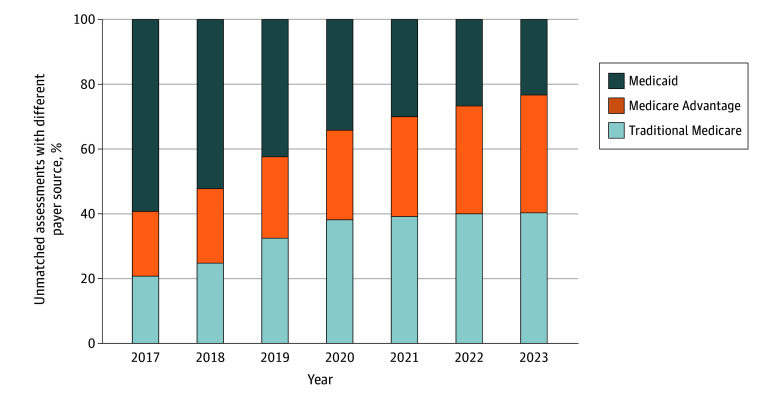
Bar Graph of OASIS Assessments Unmatched With Master Beneficiary Summary File by Year and Payer Source Absolute numbers are reported in eTable 1 in Supplement 1. OASIS assessments were attributed to a year based on the assessment effective date. The denominators include all individuals included in OASIS who were not matched to Master Beneficiary Summary File, including both Medicare and non-Medicare beneficiaries. Payer source information was based on OASIS assessments. In scenarios where multiple payer sources were noted, the decision hierarchy was Medicare fee for service, Medicare Advantage, and Medicaid. Among the OASIS assessments unmatched with MBSF, approximately 0.6% of assessments had both Medicare fee for service and Medicaid noted as the payer source and 0.7% assessments had both Medicare Advantage and Medicaid noted as the payer source.

Among unmatched assessments, the proportion with Medicaid as the payer source recorded in OASIS decreased from 59.1% in 2017 to 22.7% in 2023, while the proportion with Medicare as the payer source increased over time. In 2023, among more than 4 million unmatched assessments, 77% had Medicare payer designation (41% traditional Medicare and 36% MA) (Figure 1; eTable 1 in Supplement 1). Validation results of the payer source field in OASIS against Medicare enrollment files are available in eTable 2 in Supplement 1. OASIS payer source variables were highly concordant with MBSF enrollment for Medicare FFS and MA when records were matched. For example, in 2017, 94% of assessments with Medicare FFS as the payer source were matched to the MBSF with the same enrolled plan type in the corresponding month and 88% of assessments with MA as the payer source were matched to the MBSF with the same enrolled plan type in the corresponding month. The decreasing concordance over time was driven by increasing unmatched assessments to MBSF.

Table 2 shows counts of assessments and claims identified as belonging to FFS enrollees identified from the MBSF. Among OASIS assessments matched to the MBSF, the number of assessments matched to FFS enrollees in the MBSF decreased from 11 989 538 in 2017 to 7 207 809 in 2023, and the number of unique FFS enrollees with at least 1 assessment decreased from 3 611 412 in 2017 to 2 220 980 in 2023. This decrease is partly due to increasing MA enrollment. Over the same period, the number of FFS home health claim records increased (Table 2), partly reflecting the shortening of the payment period from 60 to 30 days with the implementation of the PDGM. Notably, the number of unique FFS beneficiaries with claims decreased from 3 424 394 in 2017 to 2 636 931 in 2023. Thus, from 2017 to 2023, the number of FFS enrollees in MBSF with a matched assessment decreased by 38.9%, whereas the number of FFS enrollees in MBSF with a claim decreased by 23.5%, suggesting an increasing incompleteness of linked home health assessments over time.

**Table 2.** Count of Matched OASIS Assessments and Home Health Claims Among FFS Medicare Beneficiaries in the Medicare Beneficiary Summary File, 2017-2023

Year	Total count of OASIS records from FFS beneficiaries^a^	Unique FFS beneficiaries with any OASIS assessments,^a^ No.	Total count of FFS home health claims^b^	Unique FFS beneficiaries with any home health claims,^b^ No.
2017	11 989 538	3 611 412	6 412 483	3 424 394
2018	11 636 198	3 504 484	6 288 214	3 379 409
2019	10 947 730	3 304 084	6 098 265	3 307 645
2020	9 425 314	2 846 645	9 232 333	3 016 570
2021	8 936 055	2 722 619	10 424 678	3 165 793
2022	8 145 070	2 497 391	8 682 501	2 874 575
2023	7 207 809	2 220 980	7 837 247	2 636 931

Abbreviations: FFS, fee-for-service; MBSF, Medicare Beneficiary Summary File; OASIS, Outcome and Assessment Information Set.

^a^ FFS was defined as the fee-for-service status based on MBSF in the month of the MBSF matched OASIS assessment or home health claims. OASIS assessments were attributed to a year based on the assessment effective date.

^b^ Home health claims record was attributed to a year based on the start date of the claim episode. The home health claims episode was defined as a single payment period (ie, 60 days prior to 2020, 30 days starting in 2020).

**Figure 2.**
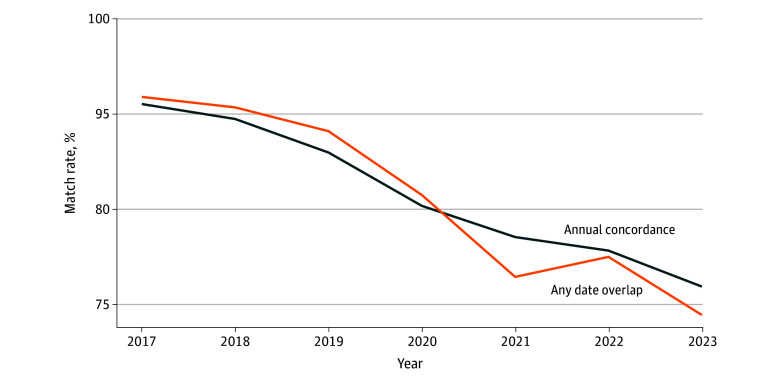
Line Graph of Match Rates Between Fee-for-Service (FFS) Claims and Outcome and Assessment Information Set (OASIS) Assessments Using Alternative Methods Annual concordance measures the percentage of FFS individuals with any home health claim who have 1 or more matched assessment in the corresponding year. We used the effective date on OASIS assessments to define a match for the unique FFS beneficiary/year. Any date overlap measures the percentage of FFS claims that can be traced with an assessment based on any start and end date overlap. We defined a match as having any day during OASIS episodes (ie, OASIS start date, OASIS end date if not missing, OASIS effective date) that can be found in the home health claims episode for the same FFS Medicare beneficiary. For both cases, we did not include the home health agency clinician identification in the match.

These patterns can also be shown by the share of claims that can be traced by an OASIS assessment using 2 matching approaches. Using approach 1, among FFS beneficiaries with at least 1 HHA claim in a given year, the share with at least 1 OASIS assessment in the corresponding year decreased from 95.6% to 76.9% (Figure 2). Using approach 2, the share of FFS HHA claims that could be matched to an assessment based on overlapping episode windows decreased from 96.8% to 73.9%. The more rapid decrease in the match rate after 2020 could be partially explained by the change in HHA payment episodes (ie, from 60 days to 30 days after 2020). However, both approaches demonstrated a decrease in matching between FFS HHA claims and OASIS.

**Figure 3.**
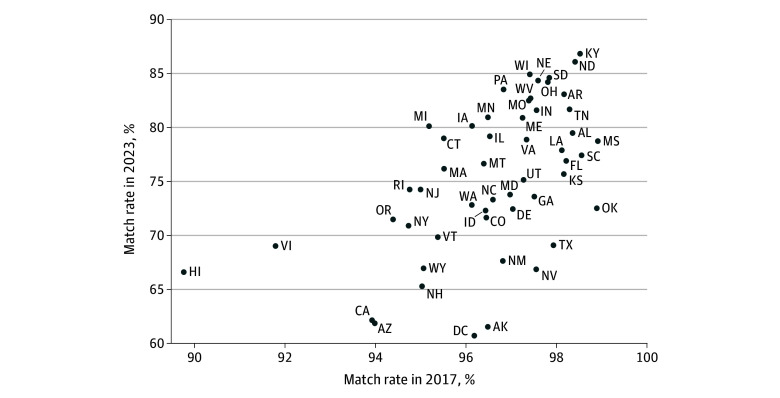
Dot Plot of State Variation in Home Health Claims–Outcome and Assessment Information Set Match Rates, 2017 vs 2023 Match rate reflects the share of fee-for-service claims that can be traced with an assessment based on any start and end date overlap. Specific numbers are reported in eTable 3 in Supplement 1. States are indicated with US postal abbreviation.

State-level match rates based on overlapping episode windows decreased from 2017 to 2023, with variation across states (Figure 3; eTable 3 in Supplement 1). While, in 2017, state match rates were all above 90%, by 2023 match rates were as low as 62% (eg, Arizona and California), and all states had match rates below 90%.

## Discussion

In this national analysis of OASIS assessments and FFS home health claims from 2017 through 2023, we found a substantial and worsening decrease in linkage completeness over the entire period. Despite stable annual volumes of OASIS assessments and assessed individuals, the share of OASIS assessments that could be linked to a Medicare beneficiary decreased from 90% in 2017 to 76% by 2023. In parallel, the proportion of FFS home health claims that could be matched to an OASIS assessment decreased to 73.4% in 2023, with even lower match rates in some states. These reductions exceed the 8% to 12% decrease in OASIS-to-beneficiary match rates noted in CCW technical documentation beginning in late 2019, suggesting that the practical impact for research that relies on linking OASIS, enrollment, and claims may be larger than what is implied by brief technical notes alone. CCW documentation further emphasizes that values received from CMS source systems are not altered or corrected, implying that inconsistent identifiers may persist in the research files and remain unresolved for end users.

These patterns may reflect a disruption in the upstream processes required to map OASIS person identifiers to Medicare beneficiary identifiers and, by extension, to support linkage between OASIS and claims. Conceptually, linkage depends on multiple steps: OASIS assessments are submitted by agencies to CMS systems (currently, the Internet Quality Improvement and Evaluation System),^14^ then processed and deidentified for release as research files through CCW/ResDAC; researchers can link those deidentified OASIS records to enrollment and claims only through encrypted beneficiary identifiers. At the claims-processing level, CMS also uses OASIS-related information reported on claims (eg, the OASIS submission date reported using occurrence code 50) to support claims adjudication, and claims may be denied when required OASIS information is missing, invalid, or does not align with clinician identifiers and beneficiary identifiers. At the same time, late or amended OASIS assessments can be accepted for up to 24 months (reduced from 36 months as of 2020) after services are rendered and claims are submitted.^15^ Taken together, these operational procedures create several points at which linkage can decrease, even if the underlying assessment and claims streams remain intact, particularly if identifier generation, identifier mapping, or deidentification steps change over time.

The timing of this linkage deterioration is particularly consequential because it coincides with major shifts in home health care delivery and policy, including implementation of PDGM in January 2020 and the COVID-19 pandemic, periods during which accurate measurement of home health use and outcomes is essential. The recent MedPAC report^20^ documented decreases in home health use based on claims data, and the number of FFS users they report aligns closely with our claims-based estimates. Several recent studies have also documented decreases in home health use using both claims and OASIS.^21^ However, our results indicate that existing OASIS-linked research files may not be able to distinguish true utilization changes from increasing underascertainment driven by linkage failures.

In the short term, investigators should interpret OASIS-based levels and trends with caution when analyses require linkage to Medicare enrollment or claims. When OASIS-based exposures are linked to claims-based outcomes, such as studies examining hospitalization using claims and functional status using OASIS^22^ or studies linking care intensity from claims with cognitive impairment from OASIS,^23^ decreasing match rates introduce classical measurement error that will attenuate estimated associations. In other settings where the outcome itself depends on linking OASIS to claims, linkage failures may further bias results. For example, a recent Office of Inspector General report^24^ found that more than half of falls observed in hospital claims for Medicare home health patients were not reported on OASIS, driven largely by missing assessments. Low match rates may have resulted in an overestimation of such values.

Even when researchers examine home health outcomes using an exogenous predictor, careful attention to linkage bias remains necessary. Studies of changes over time, including COVID-19–related changes, should avoid merging OASIS and claims when trends cannot be reliably separated from changing match rates; for example, trends in community-entry home health can be estimated using claims alone.^25^ For other cases, we need to carefully assess how the exogenous predictor is correlated with matching rates. For example, for analyses comparing states or assessing state-level policy shocks, wide cross-state variation in OASIS–claims match rates means investigators must verify whether match rates are correlated with the policy variable. Where linkage errors appear to be similar across subgroups, analyses comparing PDGM-associated changes between beneficiaries with and without dementia may be more robust than analyses of absolute changes. Comparisons of home health outcomes between FFS and MA enrollees may need to rely on payer source information recorded in OASIS, rather than merging to enrollment files, until linkage performance improves.

### Limitations

This study has limitations. First, because OASIS assessments and Medicare claims are distributed to researchers in deidentified, encrypted form, we lacked a standard identifier to validate linkage at the individual level. Second, we cannot determine whether the observed deterioration reflects changes in encrypted beneficiary identifier completeness, changes in the mapping between source identifiers and research identifiers, or other upstream system changes. Third, while we did not find evidence that either data stream is itself compromised, we cannot rule out concurrent changes in submission behavior or processing that could contribute to observed patterns. Fourth, most of our analysis was limited to FFS beneficiaries because incomplete and inconsistently available MA encounter data precluded parallel assessment of OASIS linkage for MA enrollees. Finally, although CCW documentation notes a “source system change” beginning in 2019,^16^ we cannot determine whether this change explains the observed decrease in linkage, nor whether subsequent deterioration reflects the same mechanism or additional, unobserved changes in identifier mapping, identifier generation, deidentification, or data processing. Without additional guidance or corrected encrypted beneficiary identifiers from CMS and its contractors, we have limited ability to identify the exact mechanism or assess whether it can be retroactively corrected.

## Conclusions

Ultimately, CMS must correct the crosswalk and restore high match rates between OASIS and claims. At the same time, current data limitations must be clearly communicated to the research and advocacy community. The current notification by CCW in technical guidelines is inadequate. As OASIS-based research continues to inform policy decisions on home health payment, regulation, and workforce, timely resolution of these linkage issues is essential. Further research is warranted.

## Notes

Supplement 1. **eTable 1**. OASIS Assessments Unmatched with MBSF, by Year and Payer Source **eTable 2.** Validation of OASIS Payer Source: MBSF Enrollment Type Among Matched and Unmatched Records, 2017–2023 **eTable 3**. State Variation in Proportion of FFS Home Health Claims Matched to OASIS Assessments, 2023 VS 2017

Supplement 2.
Data Sharing Statement
