# Supplementary material for: Match Rates Between Home Health Assessment and Medicare Claims Data
Source: JAMA Netw Open. 2026 Apr 2;9(4):e264788. doi: 10.1001/jamanetworkopen.2026.4788 (PMC13047462; doi:10.1001/jamanetworkopen.2026.4788)
Supplement: Supplement 1. — eTable 1. OASIS Assessments Unmatched with MBSF, by Year and Payer Source eTable 2. Validation of OASIS Payer Source: MBSF Enrollment Type Among Matched and Unmatched Records, 2017–2023 eTable 3. State Variation in Proportion of FFS Home Health Claims Matched to OASIS Assessments, 2023 VS 2017 [file jamanetwopen-e264788-s001.pdf]

Supplemental Online Content

Rahman M, Wang X, Smith JM, et al. Match rates between home health assessment and Medicare claims data. *JAMA Netw Open*. 2026;9(4):e264788.
doi:10.1001/jamanetworkopen.2026.4788

eTable 1. OASIS Assessments Unmatched with MBSF, by Year and Payer Source

eTable 2. Validation of OASIS Payer Source: MBSF Enrollment Type Among Matched and Unmatched Records, 2017–2023

eTable 3. State Variation in Proportion of FFS Home Health Claims Matched to OASIS Assessments, 2023 VS 2017

This supplemental material has been provided by the authors to give readers additional information about their work.

© 2026 Rahman M et al. *JAMA Network Open*.

eTable 1. OASIS Assessments Unmatched with MBSF, by Year and Payer Source

	Payor source of unmatched assessments					
	Medicare FFS (percent)		Medicare MA (percent)		Medicaid (percent)	
2017	396,706	21.0%	375,987	19.9%	1,115,828	59.1%
2018	519,595	24.8%	483,836	23.1%	1,094,197	52.2%
2019	823,191	32.6%	631,260	25.0%	1,070,768	42.4%
2020	1,202,311	38.3%	865,519	27.6%	1,067,897	34.1%
2021	1,444,541	39.3%	1,132,611	30.8%	1,099,268	29.9%
2022	1,551,337	40.3%	1,275,850	33.2%	1,013,911	26.4%
2023	1,693,760	40.6%	1,511,559	36.2%	945,669	22.7%

Notes: OASIS assessments were attributed to a year based on the assessment effective date. Individuals Include both Medicare Advantage enrollees and fee-for-service beneficiaries. Payer source information was based on OASIS assessments. In scenarios where multiple payer sources were noted, the decision hierarchy was: Medicare FFS, Medicare MA, Medicaid.

© 2026 Rahman M et al. *JAMA Network Open*.

eTable 2. Validation of OASIS Payer Source: MBSF Enrollment Type Among Matched and Unmatched Records, 2017–2023

Panel A: OASIS Payer Source: Medicare FFS						
Year	Yes (%)			No (%)		
	Matched to MBSF FFS	Matched to MBSF MA	Unmatched	Matched to MBSF FFS	Matched to MBSF MA	Unmatched
2017	93.8	3.0	3.2	8.5	67.8	23.7
2018	92.7	3.0	4.3	7.5	69.4	23.2
2019	90.6	2.4	7.1	6.4	70.0	23.6
2020	86.3	2.3	11.5	5.8	68.5	25.7
2021	83.4	2.5	14.1	4.9	68.5	26.5
2022	81.5	2.3	16.2	4.4	69.0	26.6
2023	78.2	2.5	19.3	4.3	67.7	28.0

Panel B: OASIS Payer Source: Medicare Advantage						
Year	Yes (%)			No (%)		
	Matched to MBSF FFS	Matched to MBSF MA	Unmatched	Matched to MBSF FFS	Matched to MBSF MA	Unmatched
2017	3.8	88.1	8.1	85.3	3.8	10.9
2018	3.4	87.3	9.3	84.3	3.9	11.8
2019	3.2	85.7	11.1	82.2	3.4	14.4
2020	3.2	82.5	14.4	77.5	3.5	19.0
2021	2.8	80.8	16.4	74.6	3.8	21.6
2022	2.6	79.8	17.6	72.9	3.7	23.4
2023	2.6	77.3	20.2	69.6	4.1	26.2

Note: MBSF FFS/MA status is determined as the enrollment in FFS or MA in the month of the effective date based on OASIS assessment.

© 2026 Rahman M et al. *JAMA Network Open*.

eTable 3. State Variation in Proportion of FFS Home Health Claims Matched to OASIS Assessments, 2023 VS 2017

state	2023			2017		
	Count of home health claims	Count of OASIS assessments with overlapping date with home health claims	Match rate	Count of home health claims	Count of OASIS assessments with overlapping date with home health claims	Match rate
AK	8368	5148	61.5%	4123	3978	96.5%
AL	144838	115110	79.5%	135488	133261	98.4%
AR	82801	68786	83.1%	70268	68980	98.2%
AZ	104300	64511	61.9%	71067	66791	94.0%
CA	1173391	729233	62.1%	615064	577677	93.9%
CO	63495	45486	71.6%	55381	53414	96.4%
CT	82009	64782	79.0%	95442	91155	95.5%
DC	9364	5685	60.7%	7599	7309	96.2%
DE	31481	22806	72.4%	22735	22061	97.0%
FL	704008	541310	76.9%	553690	543807	98.2%
GA	176538	129916	73.6%	142881	139323	97.5%
HI	8467	5639	66.6%	5089	4568	89.8%
IA	39702	31818	80.1%	37281	35839	96.1%
ID	33793	24434	72.3%	28198	27192	96.4%
IL	334528	264824	79.2%	304156	293587	96.5%
IN	112591	91859	81.6%	104322	101777	97.6%
KS	69592	52671	75.7%	51694	50743	98.2%
KY	96958	84187	86.8%	108938	107328	98.5%
LA	160518	125017	77.9%	167172	164030	98.1%
MA	230754	175787	76.2%	194890	186152	95.5%
MD	139942	103235	73.8%	102884	99774	97.0%
ME	25082	20288	80.9%	29789	28970	97.3%
MI	179398	143715	80.1%	222179	211483	95.2%
MN	61746	49976	80.9%	32351	31214	96.5%
MO	88787	73237	82.5%	92107	89702	97.4%
MS	161049	126775	78.7%	127375	125991	98.9%
MT	11960	9167	76.6%	9069	8742	96.4%
NC	191258	140202	73.3%	175992	170001	96.6%
ND	6967	5996	86.1%	4347	4278	98.4%
NE	31551	26611	84.3%	25383	24772	97.6%
NH	36615	23900	65.3%	34730	33004	95.0%
NJ	148503	110246	74.2%	141281	134213	95.0%
NM	42374	28660	67.6%	32607	31568	96.8%
NV	98330	65737	66.9%	62708	61173	97.6%
NY	297596	210984	70.9%	276794	262205	94.7%

© 2026 Rahman M et al. *JAMA Network Open*.

OH	207521	174730	84.2%	214565	209859	97.8%
OK	224591	162864	72.5%	190376	188268	98.9%
OR	55265	39504	71.5%	39834	37599	94.4%
PA	250728	209380	83.5%	231141	223817	96.8%
RI	22177	16464	74.2%	19657	18626	94.8%
SC	150579	116575	77.4%	98290	96872	98.6%
SD	8444	7143	84.6%	5790	5665	97.8%
TN	187908	153468	81.7%	149805	147246	98.3%
TX	795268	549327	69.1%	806472	789807	97.9%
UT	54690	41093	75.1%	39402	38328	97.3%
VA	191574	151109	78.9%	163333	158983	97.3%
VI	739	510	69.0%	743	682	91.8%
VT	22596	15782	69.8%	19545	18641	95.4%
WA	106537	77575	72.8%	74530	71643	96.1%
WI	64069	54402	84.9%	51788	50448	97.4%
WV	42531	35174	82.7%	44854	43700	97.4%
WY	10724	7179	66.9%	6721	6389	95.1%

* Match rate calculated following Approach 2

© 2026 Rahman M et al. *JAMA Network Open*.
