# Supplementary material for: Match Rates Between Home Health Assessment and Medicare Claims Data
Source: JAMA Netw Open. 2026 Apr 2;9(4):e264788. doi: 10.1001/jamanetworkopen.2026.4788 (PMC13047462; doi:10.1001/jamanetworkopen.2026.4788)
Supplement: Supplement 2. — Data Sharing Statement [file jamanetwopen-e264788-s002.pdf]

Data Sharing Statement

Rahman. Match Rates Between Home Health Assessment and Medicare Claims Data. *JAMA Netw Open*. Published April 02, 2026. doi:10.1001/jamanetworkopen.2026.4788

Data
Data available: Yes
Data types: Data dictionary
How to access data: We cannot share the individual-level data we accessed under a data use agreement.
When available: With publication

Supporting Documents
Document types: Statistical/analytic code
How to access documents: We put the detailed codes in a repository and the analytic code is available via the Brown Digital Repository at https://doi.org/10.26300/47qx-k924.
When available: With publication

Additional Information
Who can access the data: We put the detailed codes in a repository and shared the link in the manuscript.
Types of analyses: Not applicable
Mechanisms of data availability: Not applicable
